# Secondary Gastrointestinal Melanoma of Unknown Origin: A Case Report of a Rare Entity

**DOI:** 10.7759/cureus.4720

**Published:** 2019-05-22

**Authors:** Leon D Averbukh, Marianna G Mavilia, Amreet K Aujla

**Affiliations:** 1 Internal Medicine, University of Connecticut Health Center, Farmington, USA

**Keywords:** oculocutaneous melanoma, gastrointestinal melanoma, metastatic melanoma, pseudoachalasia, melanoma of unknown primary

## Abstract

Metastatic oculocutaneous melanoma is a malignant process most commonly identified in the lungs, bone, gastrointestinal tract (most frequently the liver), and brain. In most cases, the primary oculocutaneous lesion responsible for the metastases is identified. However, in very rare cases, patients present with metastatic lesions with an occult primary site, termed melanoma of unknown primary (MUP), secondary to the partial or complete regression of the primary lesion. We describe the case of an 89-year-old male whose initial diagnosis of achalasia was later identified to be MUP in the cardia of the stomach with protrusion into the esophagus.

## Introduction

The gastrointestinal (GI) tract, most frequently the liver, is a common site of metastases for oculocutaneous melanoma [1]. Very rarely, gastrointestinal melanoma is observed without the identification of a primary oculocutaneous lesion, termed melanoma of unknown primary (MUP). We discuss the case of an 89-year-old male with previously diagnosed achalasia who presented with worsening symptoms of dysphagia and unintentional weight loss and was subsequently found to have metastatic melanoma in the stomach cardia extending into the gastroesophageal junction (GEJ) with no identifiable primary lesion (Abstract: Leon D. Averbukh, Marianna G. Mavilia. Secondary Gastrointestinal Melanoma of Unclear Origin. Annual Scientific Meeting of the American College of Gastroenterology; October 2018).

## Case presentation

**Figure 1:**
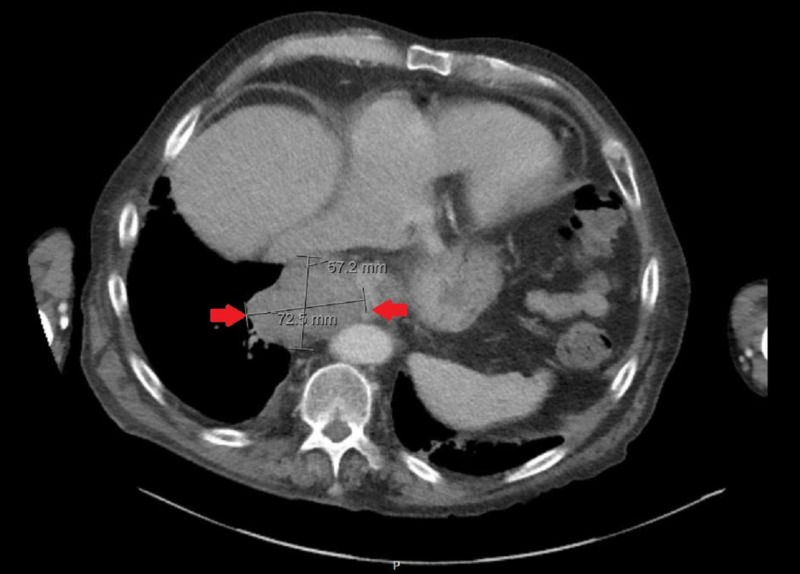
CT thorax with contrast showing 7.5-cm mass at the GE Junction (between red arrows). GE: Gastroesophageal

**Figure 2:**
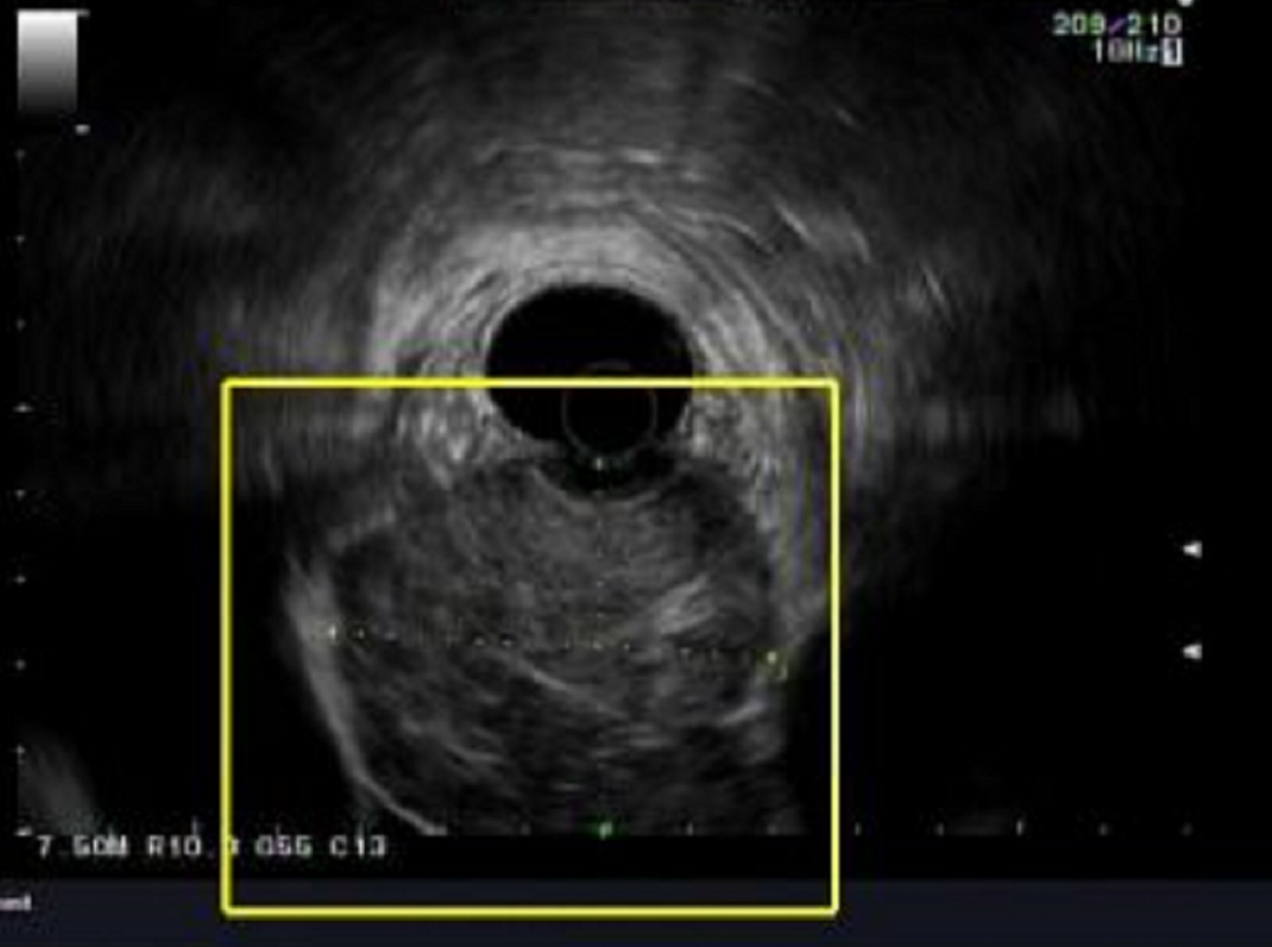
Mass measuring 68 x 55 mm abutting the gastric wall on EUS (within yellow square). EUS: Endoscopic ultrasound

**Figure 3:**
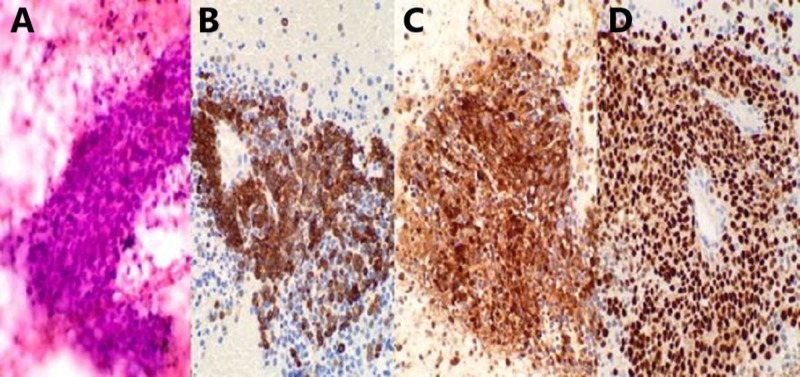
Fine needle aspiration of the perigastric mass displaying cellular smears composed of sheets or clusters of atypical epithelioid cells (A). By immunohistochemistry, neoplastic cells positive for melanoma cocktail (B), S100 protein (C) and SOX10 (D). The morphologic and immunohistochemical features are most consistent with metastatic melanoma.

An 89-year-old male with a past medical history significant for atrial fibrillation and chronic obstructive pulmonary disease presented to the emergency department with difficulty swallowing. He had been having increasing dysphagia for solids over the past year and was now unable to swallow liquids. He admitted to an unintentional 20-pound weight loss over the preceding year. Though the patient admitted to smoking in the past, he had quit in 1946. Esophagogastroduodenoscopy at an outside hospital a week prior to admission showed esophageal narrowing for which the patient received Botox injections for suspected achalasia. At the time of present admission, the patient appeared to be malnourished but was hemodynamically stable. Laboratory results were unremarkable. A contrast-enhanced computed tomography (CT) of the thorax showed a 7.5 cm mass at the GEJ (Figure 1). The patient subsequently underwent endoscopic ultrasound (EUS) which identified a lobulated mass just below the GEJ measuring 68 x 55 mm (Figure 2). Biopsy showed an epithelioid/spindle malignant tumor consistent with metastatic melanoma (Figure 3). He underwent a positron emission tomography (PET) scan which identified lesions with fluorodeoxyglucose uptake in the epicardium as well as left hilar region. No primary oculocutaneous lesion was identified on integumentary or ocular exam. The patient was deemed to be a poor surgical candidate and instead had a percutaneous endoscopic gastrostomy tube placed with plans for palliative chemotherapy.

## Discussion

Metastatic melanoma has a predilection for the lungs, bones, gastrointestinal tract, and brain [1]. Overall, the incidence of GI organ metastases in metastatic melanoma is 43%, with the liver being the most common site (58.3% of GI cases) [1]. The stomach is a much rarer metastatic destination within the GI tract: 7th overall after liver, peritoneum, pancreas, small bowel, spleen, and colon [2]. While all sub-types of melanoma can metastasize to the GI tract, the superficial spreading oculocutaneous variant is most commonly responsible. Though in a majority of cases a primary lesion is found, in an exceedingly few number of cases, thought to be roughly 2%, no primary lesion is ever identified [2]. While complete regression of primary lesions is rare, partial regression is seen in as many as 10-35% of cases and is roughly six times as likely to occur in melanoma when compared to other primary malignancies [3,4]. It is theorized that in cases of MUP, primary lesions either undergo complete regression or, at the very least, fall under detection threshold.

While MUP is an accepted pathology for GI melanomas with no identifiable oculocutaneous lesions, primary GI melanoma remains much more controversial. Few cases of the condition have been reported in literature and the mechanism behind it is not well defined [5]. Hypotheses for the disease origin include mutation of neural crest cells known to exist throughout the GI tract as well as defective ectodermal differentiation and migration of melanocytes within the GI tract [2]. Unlike metastatic melanoma tumors, primary lesions are considered more aggressive in their growth due to the rich lymphovascular supply available in the intestinal mucosa [5].

No standard guidelines have been developed to differentiate between primary and secondary GI melanoma. The identification of a single solitary tumor in the GI mucosa with additional intramucosal melanocytic lesions in the surrounding intestinal epithelium and no other cutaneous or mucosal lesions is thought to be indicative of a primary lesion [6]. In the case of our patient, the identification of multiple lesions in different organ systems on PET scan made his GI malignancy more likely to be MUP than a primary melanoma.

The prognosis of metastatic melanoma is poor, with a mean survival of only six to eight months and a five-year survival rate of less than 10% [7]. Outcomes also depend on the site of metastases, with worse survival rates in those with metastases to visceral organs, particularly the GI tract. Treatment options for metastatic melanoma in the GI tract include surgical resection, immunotherapy, and biochemotherapy. Mean survival is 48.9 months for curative resection, 5.4 months for palliative procedures, and 5.7 months for non-surgical interventions [7].

## Conclusions

MUP is an exceedingly rare type of metastatic melanoma most likely occurring secondary to either complete regression of the primary lesion or regression below detection threshold. Unfortunately, even with resolution of the primary lesion, metastatic melanoma has a poor mean survival rate, especially in those with metastatic spread to visceral organs.
